# Study of Adrenal Functions using ACTH stimulation test in Egyptian children with Sickle Cell Anemia: Correlation with Iron Overload

**Published:** 2015-04-01

**Authors:** Adel A Hagag, Mohamed S El-Farargy, Amany M Abo El-enein

**Affiliations:** 1 Pediatricsand Clinical Pathology; 2 Departments, Faculty of Medicine, Tanta University, Egypt

**Keywords:** Sickle cell anemia, Adrenal functions, Iron overload

## Abstract

**Background:** Sickle-cell anemia is characterized by defective hemoglobin synthesis with production of sickle hemoglobin. Sickle red blood cells become deformed and rigid with difficulty to pass through narrow capillaries and frequent clotting and thrombosis leading to repetitive vascular occlusions and progressive organ damage. We conducted this work to study some adrenal functions using ACTH stimulation test in Egyptian children with sickle cell anemia in correlation with iron overload.

**Methods: **This study was conducted on 60 children with sickle cell anemia who were attendants of Hematology unit, Pediatric department, Tanta university hospital in the period from April 2012 to May 2014 including 34 males and 26 females with their age ranging from 5-17 years and main age value of 13±2.9 in comparison with 30 healthy children matched for age and sex as a control group. For all patients the following were done: Complete blood count, Hb electrophoresis, serum ferritin, iron, iron binding capacity, sodium and potassium, random blood glucose, thyroid functions , morning basal cortisol levels and adrenocorticotrophic hormone stimulation test.

**Results** **: **There was significantly lower basal morning cortisol in patients than controls (mean value in patients were 8.78±3.53 ug/dl compared with 11.79±2.32 ug/dl in control group with p value of 0.021). No significant differences in adrenocorticotrophic hormone stimulation test between patients and controls were detected. (Mean value in patients were 23.078±3.709 ug/dl compared with 24.492±5.006 ug/dl in control group with p value of 0.389). Significant negative correlation was found between serum cortisol and ferritin in patients group (r= 0.625 and p value =0.003)

**Conclusion: **There was significantly lower basal morning cortisol in sickle-cell anemia patients with significant negative correlation with iron overload so regular follow up to adrenal functions to detect any adrenal impairment, as SCD patients are vulnerable to adrenal hypo function, is recommended.

## Introduction

 Sickle-cell disease anemia is an inherited hemoglobin disorder characterized by defective hemoglobin synthesis with production of an abnormal form of hemoglobin, known as sickle hemoglobin (Hb S).^1^

 HbS is caused by mutation in β-globin gene in which the 17^th^ nucleotide is changed from thymine to adenine and the sixth amino acid in the β-globin chain becomes valine instead of glutamic acid; this disrupts RBCs architecture and flexibility and promotes cellular dehydration, with physical and oxidative cellular stress.^2^

 Endocrine and metabolic disorders may occur in sickle cell anemia and have multifactorial causes including tissue hypoxia, chronic anemia and iron overload. Endocrinal manifestations include growth delay, osteopenia and hypogonadism, diabetes mellitus, thyroid and adrenal disorders.^3^ Patients with sickle cell disease have higher risk of developing adrenal insufficiency than the general population.^4^

**Aim of the work**

 The aim of this work was to study adrenal functions using basal morning cortisol levels and ACTH stimulation test in children with sickle cell anemia in correlation with iron overload.

**SUBJECTS AND METHODS**

   This study was done after approval from ethical committee of research center of Tanta University and written consent from the parents of all children included in this study and was conducted on 60 children with sickle cell anemia (Hb SS) with serum ferritin more than 1000 ng/ml under follow up in Hematology unit, Pediatric department, Tanta University in the period from April 2012 to May 2014 including 34 males and 26 females with their age ranging from 5-17 years and main age value of 13±2.9 years and 30 healthy children as a control group including 16 males and 14 females with their ages ranging from 7-17 years and mean age value of 11.7±3.19 years. 

**Exclusion criteria**                    

 Children who received hormonal treatment. 

**All patients and control groups were subjected to the following**

**a) Complete history taking: **with special account on manifestations of insufficient cortisol production as muscle weakness, fatigue and increased skin pigmentation and transfusion regimen.

**b) Thorough clinical examination with special account on:** Anthropometric measurements: weight and height, pallor, jaundice, splenomegaly, hepatomegaly and manifestations of adrenal dysfunctions.

**Laboratory investigations**

**Specimen collection and handling**

 Five ml of venous blood were collected using sterile needles through gentle venipuncture after sterilization of puncture site by alcohol, and collected samples were divided into; 2 ml was delivered on 20 uL EDTA solution for complete blood count using ERMA PCE-210 N cell –counter ^5^ and hemoglobin electrophoresis, ^6^ the rest of blood was put in a plain tube that was allowed for clotting and then centrifugation at 1500x for 10 minutes was performed. Separated serum was used for assessment of serum ferritin, serum iron and total iron binding capacity, serum sodium, serum potassium, random blood glucose, thyroid functions, basal morning cortisol levels and adrenocorticotrophic hormone (ACTH) stimulation test.

**Determination of serum iron status**

 Including serum iron according to procedure recommended from Biomaghreb company,^7^ serum total iron binding capacity (TIBC) according to procedure recommended from Biomaghreb company^8^ and serum ferritin levels by ELIZA [DRG® Ferritin ELISA (EIA-4292)].^9^

**Determination of basal serum cortisol levels and ACTH stimulation test**

 Baseline cortisol was determined by electrochemiluminescence immunoassay “ECLIA” using Elecsys and cobas immunoassay analyzers;^10^ then ACTH (synactin or cosyntropin) 5 µg/kg IV or IM was injected to the patients and 1ml blood was collected after 1 hour and allowed for complete clotting. Centrifugation then was done and separated serum was used to measure cortisol level**.** Rise in cortisol level to more than 18 ug/dl within 60 minutes demonstrates normal results while rise to less than 18 ug/dl demonstrates an abnormal response. If the adrenal glands were not adequately stimulated in response to ACTH, the patient has suppressed adrenal glands. An elevated baseline ACTH level suggests primary adrenal failure.^11^

**Determination of thyroid function tests**

 Including Free T_3,_ Free T_4 _and TSH.^12^

**Statistics**

 Statistical presentation and analysis of the present study was conducted, using the mean, standard error, student t- test, Chi-square, Linear Correlation Co*e*fficient tests by SPSS V17.

## Results

 There were no significant differences between patients and controls regarding age, sex, but there were significant differences between patients and controls regarding family history, consanguinity, weight, height and body mass index (BMI) with higher consanguinity rates and positive family history and lower body weight, height and BMI in patients compared with control group Table 1. 

**Table 1:** Demographic data and anthropometric measurements in patients and controls

	**Patients (no=60)**	**Controls (no=30)**	** X** ^2^ ** P-value**	
**Age (Years) ** **Range ** **Mean ±SD**	5-17 13±2.90	7-17 11.7±3.199	1.119	0.273
**Sex ** **Males ** **Females**	32 (53.33%) 28 (46.6)	16 (53.33%) 14 (46.6)	0.068	0.794
**Family history of SCA** **Positive** **Negative**	26 (43.3%) 34 (56.6%)	- 30 (100%)	21.538^ X2^	<0.001*
**Consanguinity** **Negative** **Positive**	20 (33.33%) 40(66.66%)	24(80%) 6 (20%)	8.552	0.003*
**Weight (kg)** **Range ** **Mean ±SD**	21-59 41.1±10.686	26-60 49.8±9.807	2.158	0.039*
**Height (cm) ** **Range ** **Mean ±SD**	121-156 140±10.705	126-165 149±5.195	2.361	<0.025*
**BMI (%)** **Range ** **Mean ±SD**	11.7-20 15.30±2.72	13.5-23.6 18.09±3.56	-2.776 ^t^	0.008*

* Significant: P<0.05. SCA: Sickle cell anemia, BMI: Body mass index

 Pallor and jaundice were the most common presenting symptoms while hepatomegaly and splenomegaly were the most common presenting signs in patients group and most of studied patients received blood transfusion every more than 4 weeks (Table 2). The age of 1^st^ transfusion in studied patients ranged from 9-72 months with mean age of first transfusion of 24 ± 13.081 months and interval of transfusion ranged from 2- 28 weeks with mean interval of 4.09 ± 2.652 weeks (Table 2).

**Table 2:** Clinical data in studied patients

**Clinical data**	**Number of patients (%)**
**Pallor** **Jaundice ** **Hepatomegaly ** **Splenomegaly** **Autosplenectomy** **Age of 1** ^st^ ** transfusion** **in months** : **Range** **(mean)** **Frequency of blood transfusion** **Every 2 weeks** **Every 3-4weeks** **Every more than 4 weeks** **Interval of transfusion in weeks:** **Range** **(mean) Skin pigmentations** **Cholecystectomy** **Leg ulcer** **Sickle cell crisis ** **Stroke**	54 (90%) 39 (65%) 60 (100%) 24 (40%) 36 (60%) 9 -72 (24 ± 13.) 6 (10%) 12 (20%) 42 (70%) 2 -28 (4.09 ± 2.65) 12 (20%) 6 (10%) 6 (10%) 18 (30 %) 3 (5 %)

 There were significantly lower red blood cells (RBCs), hemoglobin (Hb), and significantly higher reticulocytes, platelets and white blood cells (WBCs) in patients than control group and no significant differences in mean corpuscular volume (MCV) and mean corpuscular hemoglobin (MCH) between patients and control group (Table 3).

 There were significantly lower total iron binding capacity, mean serum sodium and basal morning serum cortisol levels and significantly higher serum ferritin, iron, potassium in patients than control group and no significant differences between patients and control group regarding random blood glucose and adrenocorticotrophic hormone stimulation test (Table 4).

**Figure 1:**
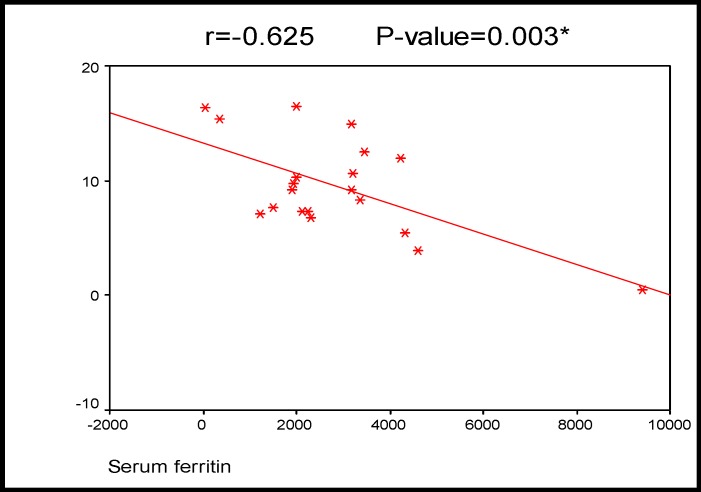
Correlation between serum ferritin and cortisol levels in patients group

There was significant negative correlation between serum ferritin and serum cortisol levels in SCD patients (Table 5 and Figure 1). 

 There were no significant differences in thyroid hormones (FT3, FT4 and TSH) between patients and controls but hypothyroidism was detected in three patients (5%) (Table 6).

**Table 3:** Comparison between patients and controls regarding CBC parameters

	**Patients ** **(no=60)**	**Controls ** **(no=30)**	**X** ^2^ ** P-value**	
**RBCs ** **(million cell/mm³** **) ** **Range ** **Mean ± SD**	2.3-3.5 2.70 ± 0.69	4.1-4.5 4.35 ± 0. 55	10.78	<0.001*
**Hb (g/dl)** **Range ** **Mean ± SD**	6.3-10 7.945±0.92	11.4-12.4 11.9±0.31	-17.381	0.000*
**MCV (fl)** **Range ** **Mean ± SD**	77.5-93 80.6±.83	82.9-95 88.39±10.88	1.976	0.058
**MCH (pg)** **Range ** **Mean ± SD**	27-30 27.25±4.34	27.4-32 29.96±5.51	1.474	0.152
**WBCs ** **(cells/mm³**) **Range ** **Mean ± SD**	8-20 14.9±3.291	7-10 9.34±1.34	6.195	<0.001*
**Platelets (thousands/mm** ^3^ **)** **Range ** **Mean ± SD**	160-688.8 359.6±32.4	150-430 292±18.75	**-**3.5	0.030*
**Reticulocytes (%)** **Range ** **Mean ± SD**	1.2-7.2 4.095±1.723	0.5-1.5 0.920±0.326	7.962	<0.000*

* **Significant** (P<0.05), **CBC: **Complete blood count, **RBCs:** Red blood cells, **Hb:** Hemoglobin, **MCV:** Mean corpuscular volume, **MCH:** Mean corpuscular hemoglobin, **WBCs**: White blood cells

**Table 4:** Comparison of Na, potassium, random sugar, basal morning cortisol, ACTH stimulation test and serum iron status between patients and controls

**Parameters**	**Patients (no=60) **	**Controls (no=30)**	**t **	**P value**
**Serum sodium (mEq/L)** **Range ** **Mean± SD **	105-148 131.15±8.55	135-144 137.80±3.32	-2.354	0.025*
**Serum potassium** ** (mg/dl)** **Range ** **Mean± SD**	4.57-5.98 5.24±.0.69	3.5-4.5 4.02± 0.32	0.691	0.049*
**Random blood sugar** ** (mg/dl)** **Range ** **Mean± SD**	52 -125 89.35±24.618	92-115 105.3±8.505	1.976	0.058
**Cortisol AM (** **ug** ** /dL) ** **Range ** **Mean± SD**	0.47-16.4 8.78±3.53	7.3-14.3 11.79±2.32	-2.432	0.021*
**ACTH stimulation test (** **ug/dl** **) ** **Range ** **Mean± SD**	21-38 23.078±3.709	21-38 24.492±5.006	0.876	0.389
**Serum ferritin (ng/ml)** **Range ** **Mean± SD**	1022-9424 2683±1947	123-305 193.3±66.82	6.11	0.000*
**Serum iron (ug/ml)** **Range ** **Mean± SD**	155-349 158.9±84.582	71-100 82.3±8.564	4.009	0.001*
**Serum TIBC (ug/ml)** **Range ** **Mean± SD**	165-332 234.85±39.917	263-311 288.6±16.621	-5.189	0.000*

* **Significant**, **TIBC**: total iron binding capacity, **AM**: Morning or Ante Meridiem**,** **Na**: Sodium. **ACTH** **stimulation** **test**: Adreno corticotrophic hormone stimulation test

**Table 5:** Correlation between serum iron, serum ferritin and serum sodium, potassium, random blood glucose, basal cortisol and ACTH stimulation test.

	**Serum iron**		**Serum ferritin**		**TIBC**	
**Correlations**	** r**	**P-value**	** r**	**P-value**	** r**	**P-value**
**Serum sodium**	-0.601	0.005*	-0.589	0.006*	0.591	0.006*
**Serum potassium**	0.124	0.601	0.182	0.443	0.200	0.398
**Random blood glucose**	0.077	0.747	0.264	0.261	0.243	0.301
**Cortisol AM**	-0.461	0.041*	-0.625	0.003*	0.530	0.016*
**ACTH stimulation test**	0.262	0.265	0.039	0.869	0.127	0.593

* **Significant, AM:** Morning or Ante Meridiem, **TIBC:** Total iron binding capacity, **ACTH stimulation test:** Adrenocorticotrophic hormone stimulation test

## Discussion

 Sickle cell disease is an inherited disorder, characterized by defective hemoglobin synthesis with production of Hb S. Red blood cells that contains Hb S become deformed and rigid. This, in turn, impedes their ability to pass through narrow capillaries, with frequent clotting and thrombosis leading to repetitive vascular occlusions and progressive organ damage.^1^

 The aim of this work was to study adrenal functions using basal morning cortisol levels and ACTH stimulation test in children with sickle cell anemia in correlation with iron overload.

 In this study, there were significantly higher serum ferritin and serum iron and significantly lower TIBC levels in patients than control group. This is in agreement with Patra and Khodiar (2012)^13^ and Akinsegun et al. (2013)^14^ who found the same results and explained this by chronic hemolysis and repeated blood transfusion.

 In the present study, there was significantly lower serum sodium and significantly higher serum potassium levels in patients than control group. This is in agreement with Pandey et al. (2012)^15^ who found the same results and attributed this to renal insufficiency or hemolytic crises.

 In this study, there was no significant difference between patients and controls regarding random blood glucose. This is in agreement with Mohapatra (2005)^16^ who found rare occurrence of diabetes mellitus (either type1 or type 2) in SCD patients and stated that SCD patients in tropics seem to enjoy relative protection from diabetes. 

Theoretical theories for such protection would include the low BMI, hyper metabolism and possibly other genetic factors.

 In the present study, there were significantly lower mornings basal serum cortisol levels in patients compared with control group, and no significant difference between patients and controls as regard ACTH stimulation test. This is in agreement with Smiley et al. (2008)^17^ who found cortisol deficiency in patients with sickle cell anemia and attributed this cortisol deficiency to many factors as iron overload, vascular insufficiency or hypothalamic pituitary adrenal axis affection , Soliman  et al. (1995)^18^ who suggested that the main cause of cortisol deficiency is HPA axis affection, due to hypoxic-vascular insults to hypothalamic pituitary axis during one or more of sickling episodes, Lawrence (2012)^19^ who found low serum cortisol level in SCD patients compared with controls and normal response to ACTH stimulation test with increased serum cortisol within 1 hour and Saad (1992)^20^ who found no significant difference between pediatric SCD patients and control cases as regard ACTH stimulation test.

 In this study there were no significant differences in thyroid hormones (FT3, FT4 and TSH) between patients and controls but hypothyroidism was detected in three patients (5 %); this is in agreement with Rhodes et al. (2009)^21^ who found no statistically significant differences between patients with SCA and controls in levels of thyroid hormones and Ozen et al. (2013)^22^ who found that hypothyroidism was detected in 3 patients (6%) in a 

study that included 50 patients with SCA. On the contrary, El-Sarraf et al. (2009)^23^ found significant lower T4 level in SCD patients compared to controls**.**

**Table 6:** Comparison between patients and controls as regard thyroid hormones

**Thyroid ** **hormones**	**Patients ** (no=60)	**Control ** (no=30)	**P value**
**TSH** Range Mean ± SD	1.12-5.7 2.31±1.2	1.2-2.89 2.01±0.54	0.45
**FT3** Range Mean ± SD	2.5-4 3.81±1.3	2.65-4 3.33±0.47	0.27
**FT4** Range Mean ± SD	0.79-1.82 1.37±0.274	1.1-1.65 1.34±0.164	0.57

**TSH:** thyroid stimulating hormone, **FT3:** Free triiodothyronine, **FT4:** Free thyroxine

## CONCLUSION

 There was significantly lower basal morning cortisol in patients with sickle-cell anemia with significant negative correlation with iron overload so regular follow up to adrenal functions to detect any adrenal impairment, as SCD patients are vulnerable to adrenal hypo function, is recommended.
